# MicroRNA‐3061 downregulates the expression of PAX7/Wnt/Ca^2+^ signalling axis genes to induce premature ovarian failure in mice

**DOI:** 10.1111/cpr.13686

**Published:** 2024-06-03

**Authors:** Te Liu, Yichao Wen, Zeyu Cui, Haiyang Chen, Jiajia Lin, Jianghong Xu, Danping Chen, Ying Zhu, Zhihua Yu, Chunxia Wang, Bimeng Zhang

**Affiliations:** ^1^ Shanghai Geriatric Institute of Chinese Medicine Shanghai University of Traditional Chinese Medicine Shanghai China; ^2^ Department of Gynaecology Jingan Hospital of Traditional Chinese Medicine Shanghai China; ^3^ Department of Reproductive Medicine Henan Province Hospital of Traditional Chinese Medicine Henan China; ^4^ Department of Acupuncture, Shanghai General Hospital Shanghai Jiao Tong University School of Medicine Shanghai China

## Abstract

The in‐depth mechanisms of microRNA regulation of premature ovarian failure (POF) remain unclear. Crispr‐cas9 technology was used to construct transgenic mice. The qPCR and Western blot was used to detect the expression level of genes. H&E staining were used to detect ovarian pathological phenotypes. We found that the expression levels of microRNA‐3061 were significantly higher in ovarian granulosa cells (OGCs) of POF mouse models than in controls. The miR‐3061^+/−^/AMH‐Cre^+/−^ transgenic mice manifested symptoms of POF. RNA‐Seq and luciferase reporter assay confirmed that the PAX7 was one of the target genes negatively regulated by microRNA‐3061 (miR‐3061–5p). Moreover, PAX7 mediated the expression of non‐canonical Wnt/Ca^2+^ signalling pathway by binding to the motifs of promoters to stimulate the transcriptional activation of Wnt5a and CamK2a. In contrast, specific knock‐in of microRNA‐3061 in OGCs significantly downregulated the expression levels of PAX7 and inhibited the expression of downstream Wnt/Ca^2+^ signalling pathway. We also discerned a correlation between the expression levels of mRNAs of the Wnt/Ca2+ signalling pathway and the levels of E2 and FSH in POF patients by examining gene expression in the follicular fluid‐derived exosomes of women. We confirmed that overexpression of microRNA‐3061 induced proliferative inhibition of OGCs and ultimately induced POF in mice by suppressing the transcription factor PAX7 and downregulating expression levels of its downstream Wnt/Ca^2+^ signalling pathway genes.

## Graphical abstract

We confirmed that overexpression of microRNA‐3061 induced cell‐cycle arrest and proliferative inhibition of OGCs and ultimately induced POF in mice by negatively regulating the expression of the transcription factor PAX7 and downregulating the transcriptional activity and expression levels of its downstream Wnt/Ca2+ signalling pathway genes.

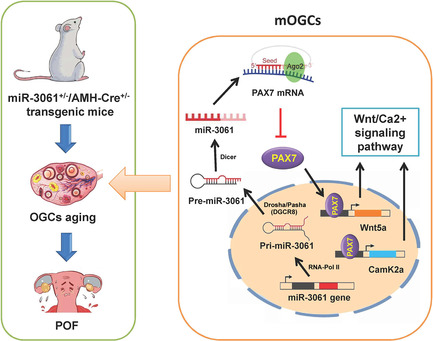

## 1 INTRODUCTION

The number of infertile women has continued to increase in recent years, and the apparent dichotomy between pathologic reproductive aging and reproductive needs has become increasingly evident; therefore, achieving an early diagnosis and treatment of female reproductive aging is key to improving the quantity and quality of births.^1^, ^2^, ^3^, ^4^, ^5^ Premature or primary ovarian insufficiency (POI) is the most common female reproductive pathology with aging, but when this stage is attained, a majority of women are already in the final stages of ovarian failure (i.e., premature ovarian failure [POF]), and this situation is no longer amenable to intervention.^1^, ^2^, ^3^, ^4^, ^5^ It is therefore important to explore the genetic aetiology of POI/POF for diagnosis, timely fertility counselling and long‐term reproductive health management.^1^, ^2^, ^3^, ^4^, ^5^ POF can be diagnosed in women before the age of 40 if they have diminished ovarian reserve, decreased follicular number with attenuated oocyte quality, amenorrhea or scanty menstruation, infertility and/or elevated pituitary gonadotropin concentrations (peripheral follicle‐stimulating hormone (FSH) >40 U/L).^3^, ^4^, ^6^ The pathogenesis of POF is very complex, the course of the disease is long and studies have shown that genetic and hereditary factors, infections and immune disorders, radiotherapy toxicity, psychological stress, endocrine disorders, chronic inflammation, environmental factors and intestinal flora dysbiosis can all lead to POF.^1^, ^2^, ^3^, ^4^, ^7^, ^8^ The ovarian follicle is the basic unit of the female reproductive system, consisting of ovarian theca cells, ovarian granulosa cells (OGCs) and the oocyte; and its normal development is essential for the maintenance of the female reproductive process.^9^, ^10^, ^11^ Although the number of follicles in the primordial follicular pool of female mammals is very large, only an exceedingly small number of follicles (approximately <1%) can develop into mature follicles and achieve ovulation. The ultimate fate of the vast majority of primordial follicles is therefore degeneration,^1^, ^2^, ^3^, ^4^, ^5^ with most OGCs destined to atresia.^1^, ^2^, ^3^, ^4^, ^5^ While it is widely recognized that the aging and death of OGCs are the chief triggers for the development of follicular atresia and POI/POF,^1^, ^2^, ^3^, ^4^, ^5^ the underlying regulatory mechanisms remain unclear. Therefore, it is of paramount importance to explore and elucidate the precise regulatory mechanisms of aging and death of OGCs so as to maintain female fertility.

A growing number of studies have indicated that epigenetic modifications—particularly aberrant microRNA regulation—are closely associated with the development of POF. MicroRNAs are single‐stranded, non‐coding, small‐molecule RNAs with a length of approximately 21–23 bases that are processed by the enzyme Dicer from a single‐stranded RNA precursor with a hairpin structure of approximately 70–90 bases in size.^12^, ^13^, ^14^ The mature microRNAs have a 5′ phosphate group and a 3′ hydroxyl group and negatively regulate the expression of target mRNAs by complementary binding to the 3′ UTR of target mRNAs, destabilizing the target mRNAs and inhibiting their translation.^12^, ^13^, ^14^ It has been shown that knockdown of Dicer1 expression in female rats leads to atypical follicular hypoplasia and infertility, suggesting that blocking microRNA processing and maturation significantly affects ovarian function.^15^, ^16^, ^17^ Dang et al. characterized the differential expression of plasma free microRNAs in 140 POF patients and found that the expression levels of miR‐22‐3p were significantly lower in POF patients than in the healthy population. It was also preliminarily confirmed that decreased miR‐22‐3p was significantly correlated with decreased ovarian reserve and POF symptoms.^15^ Ai et al. reported that tretinoin polyglycolide induced POF in mice by stimulating the expression of endogenous miR‐15a, inhibiting the activation of the Hippo‐YAP/TAZ signalling pathway and promoting the senescence and apoptosis of mouse OGCs (mOGCs).^16^ In addition, numerous studies have revealed that miR‐23a, miR‐146a, miR‐146b‐5p, miR‐144‐5p and miR‐15b engendered POF by regulating the activity of OGCs.^17^, ^18^ Our previous study also showed that cyclophosphamide attenuated the autophagic function of mOGCs by upregulating the expression of miR‐15b, thus silencing the expression of endogenous klotho mRNA and stimulating the activity of the downstream TGFβ1/Smad pathway, ultimately inducing POF in mice.^11^ A high‐sugar and high‐fat diet induced aging and POF‐like symptoms in mOGCs by inhibiting the expression of endogenous miR‐146b‐5p, activating the Dab2ip/Ask1/p38/Mapk signalling pathway and promoting the phosphorylation of γH2A.X.^19^ In addition, thymopentin promoted the transcriptional activation of Lin28A by stimulating the expression of the transcription factor YY2, inhibiting the activity of microRNAs of the Let‐7 family, alleviating pathological aging of mOGCs and ultimately ameliorating the symptoms of POF in mice.^20^ Although some microRNAs have been demonstrated to be closely associated with POF development, the regulatory mechanisms involved still require elucidation.

Based on the existing evidence, we herein screened and constructed Mir3061^+/−^/AMH‐Cre^+/−^ transgenic mice with specific knock‐in of mOGCs by systematically analysing the differential microRNA expression profile in mOGCs from a cyclophosphamide‐induced POF mouse model, in an attempt to elucidate its effect on inducing pathological aging of OGCs via an underlying regulatory mechanism.

## 2 MATERIALS AND METHODS

### 2.1 Establishment and characterization of transgenic mice

In brief, we adopted CRISPR/Cas9 technology to obtain Amh‐Cre knock‐in mice C57BL/6J‐Amh^em (2A‐Cre)2Smoc^ (Amh‐e[2A‐Cre] 2, AMH‐Cre^+/−^) and Rosa26 locus‐sited Mir3061 conditional knock‐in overexpressing mice C57BL/6J‐Gt (ROSA)26Sor^e m(EF1a‐Mir3061)1Smoc^ (R26‐e[EF1a‐Mir3061]1, miR‐3061^fl/fl^). The above two types of mice were subsequently crossed and artificially bred to obtain miR‐3061^+/−^/AMH‐Cre^+/−^ positive heterozygous conditional knock‐in mice and miR‐3061^fl/fl^ mice were used as controls. The above transgenic mice were constructed, mated and characterized by Modelorg (Shanghai Model Organisms Company, Shanghai, China). All experiments were conducted upon approval by the Ethics Committee for Animal Experiments of Modelorg.

### 2.2 Isolation and culture of mOGCs

Briefly, and in accordance with previous studies,^5^, ^8^, ^11^, ^19^, ^20^ 10‐week‐old female C57BL/6 mice were obtained from the Experimental Animal Center of the Shanghai University of Traditional Chinese Medicine, and experiments were conducted upon approval by the Ethics Committee for Animal Experimentation of our institution. The mice were necropsied after cervical dislocation, and the ovarian tissues were isolated in a sterile environment and placed in PBS at 4°C. Ovarian tissues were clipped and digested by adding 2.0 mL of hyaluronidase (0.1%, Sigma‐Aldrich, St. Louis, MO, USA) at 37°C for 1 min. The tissue suspension was fanned gently, and digestion was terminated by adding 200 μL of foetal bovine serum (Gibco, Gaithersburg, MD, USA) and filtering through a 200‐mesh cell sieve. The filtrate was mixed with 5.0 mL of PBS and centrifuged at 1500 r/min for 5 min at 10°C. The supernatant was then discarded, and the precipitate was resuspended with 5.0 mL of PBS and centrifuged at 1500 r/min for 5 min at 10°C. The supernatant was again discarded, and the cellular precipitates were resuspended and mixed with DMEM:F12 (1:1) medium containing 10% foetal bovine serum, 10 ng/mL basic fibroblast growth factor (bFGF), 10 ng/mL epidermal growth factor (EGF), 2 mM l‐glutamine, 10 ng/mL growth hormone (GH) and 15 ng/mL estradiol (E2) (all of the above reagents were purchased from Gibco, Gaithersburg, MD, USA). Cells were ultimately inoculated in six‐well cell culture plates at 37°C with 5% CO_2_ in compressed air and high humidity until 80% confluency was attained.

### 2.3 Establishment of POF cell and animal models

Congruent with previous studies,^8^, ^11^, ^19^ wild‐type C57BL/6 female mice approximately 5 weeks of age were obtained from the Experimental Animal Center of the Shanghai University of Traditional Chinese Medicine and placed on the experiment after approval by the Ethics Committee for Animal Experiments of our institution. All mice were reared in the same environment until 7 weeks of age and then injected intraperitoneally (i.p.) with 70 mg/kg cyclophosphamide (CTX, Sigma‐Aldrich) once per day for 7 consecutive days. Subsequently, 30 mg/kg CTX was injected i.p. once per day for 14 consecutive days to construct the POF mouse model. Control mice were injected with equivalent doses of saline.

According to a previous study,^8^ we cultured mOGCs in vitro until the exponential growth phase was reached by adding 38.7 μM CTX at an IC50 concentration, and we continued to culture them for 24 h to allow the creation of an in vitro cellular model of POF. Control cells were provided an equivalent volume of DMSO and cultured in an identical environment.

### 2.4 CCK‐8 assay

Briefly, the number of cells in each group was adjusted, and 2000/mL cells were inoculated into 96‐well cell culture plates.^8^ After 24 h of incubation, 10 μL of CCK‐8 solution (Sigma‐Aldrich Chemical) was added to each group of cells, and the reaction was incubated at 37°C for 3 h. The cell culture plates were placed in an enzyme marker solution, and the absorbance values were recorded at a wavelength of 450 nm. We calculated the cellular proliferation rate (%) as (the OD value of the experimental group cells/OD value of the control group cells) × 100%.

### 2.5 PI staining and flow cytometric identification

In brief, 2 × 10^5^/mL cells were collected and added to 1 mL of 70% pre‐cooled ethanol for 48 h of fixation.^8^, ^11^ The cells were centrifuged at 1500 r/min for 5 min at 4°C, the cell precipitate was collected, PI staining solution (Sigma Chemicals) was added and the reaction was performed for 30 min at 4°C protected from light. We then analysed the cell‐cycle distribution of each group of cells using flow cytometry (Cytomics FC 500, BECKMAN) and analysed the data using FlowJo software.

### 2.6 microRNA‐specific reverse‐transcription reaction and quantitative polymerase chain reaction (miR‐qPCR)

Following the steps in the instruction manual for the RNAprep pure Tissue Kit (TIANGEN Biotech Co., Ltd., Beijing, China), we removed approximately 20 mg of tissue specimen, added 800 μL of lysis solution and processed the tissue with grinding homogenization. We aspirated the supernatant, added 200 μL of chloroform, inverted and mixed the tube, centrifuged at 13,400 × *g* for 15 min at 4°C and aspirated the supernatant. We then added liquid anhydrous ethanol at twice the volume of the supernatant, inverted and mixed well again and centrifuged at 13,400 × *g* for 30 min at 4°C. Then, 75% ethanol (500 μL) was added to resuspend the RNA precipitate and the mixture was centrifuged at 13,400 × *g* for 5 min at 4°C. Excess liquid was removed, and the precipitate was added to 300 μL of DECP water for full dissolution. The purity and total concentration of the RNA were determined by taking 1 μL of RNA solution and determining the OD260/OD280 ratio (generally controlled between 1.8 and 2.0). Following the steps in the instruction manual for the miRcute microRNA First‐strand cDNA kit (TIANGEN Biotech Co., Ltd., Beijing), 20 μL (100 ng/μL) of tRNA, 25 μL of 2 × microRNA RT reaction buffer (100 ng/μL), 25 μL of 2 × microRNA RT reaction buffer, 4 μL of 1 × microRNA RT Enzyme Mix and 6 μL of RNase‐free deionized water were mixed thoroughly. The PCR cycling conditions were as follows: 42°C for 60 min for microRNA plus Poly A reaction and the reverse‐transcription reaction; followed by 95°C for 3 min for the enzyme‐inactivation reaction.

Following the protocol in the instruction manual of the miRcute microRNA qPCR Detection kit (TIANGEN Biotech Co., Ltd. Beijing), reagents and samples to be tested were added as follows: 10 μL of 2 × miRcute Plus microRNA Premix (with SYBR), 1 μL each of 1 × forward primer and reverse primer (10 μM), 4 μL of microRNA first‐strand cDNA and 10 μL of deionized cDNA were added to the real‐time fluorescence quantitative PCR instrument. The following reactions were then conducted for 40 cycles: 15 min at 95°C, 20 s at 94°C and 34 s at 60°C. The fluorescence values were then read. The primers of PCR were follows: miR‐7082‐F: 5′‐GCCCCCCTTGCTGTCTGTCTCC‐3′, miR‐7082‐R: 5′‐GCTGTCAACGATACGCTACCTA‐3′, miR‐1902‐F: 5′‐AGAGGTGCAGTAGGCATGACTT‐3′, miR‐1902‐R: 5′‐GCTGTCAACGATACGCTACCTA‐3′, miR‐22‐F: 5′‐AAGCTGCCAGTTGAAGAACTGT‐3′, miR‐22‐R: 5′‐GCTGTCAACGATACGCTACCTA‐3′, miR‐3061‐3p‐F: 5′‐CTACCTTTGATAGTCCACTGCC‐3′, miR‐3061‐3p‐R: 5′‐GCTGTCAACGATACGCTACCTA‐3′, miR‐3061–5p‐F: 5′‐CAGTGGGCCGTGAAAGGTAGCC‐3′, miR‐3061–5p‐R: 5′‐GCTGTCAACGATACGCTACCTA‐3′, miR‐1931‐F: 5′‐ATGCAAGGGCTGGTGCGATGGC‐3′, miR‐1931‐R: 5′‐GCTGTCAACGATACGCTACCTA‐3′, miR‐7013‐F: 5′‐TATGAAGAGGCCAGTGTTGTAG‐3′, miR‐7013‐R: 5′‐GCTGTCAACGATACGCTACCTA‐3′, miR‐344‐2‐F: 5′‐TGATCTAGCCAAAGCCTGACTGT‐3′, miR‐344‐2‐R: 5′‐GCTGTCAACGATACGCTACCTA‐3′.

### 2.7 Extraction of total RNA and quantitative polymerase chain reaction (qPCR)

Total RNA was extracted from each group of cells according to the procedure described in the trizol reagent manual, and the corresponding cDNA was generated with RT‐PCR using a ReverTra Ace‐α first strand cDNA synthesis kit (TOYOBO) and the oligonucleotide primer Oligo(dT)18. cDNA obtained from the above reverse transcription was used as a template and processed on an Eppendorf RealPlex4 (Eppendorf Co., Ltd.) using the SYBR Green Real‐Time PCR Master Mix (TOYOBO). The qPCR was completed with 40 amplification cycles as follows: denaturation at 95°C for 15 s, annealing at 58°C for 30 s and primer‐template extension at 72°C for 42 s. The relative gene expression levels were calculated using the 2^−ΔΔCt^ method (ΔCt = Ct_genes−Ct_18s RNA; ΔΔCt = ΔCt_all_groups−ΔCt_control_group). The mRNA expression levels were normalized to the expression level of 18 s rRNA. The primers of PCR were follows: p16‐F: 5′‐GCTCAACTACGGTGCAGATTC‐3′, p16‐R: 5′‐GCACGATGTCTTGATGTCCC‐3′, p21‐F: 5′‐CCTGGTGATGTCCGACCTG‐3′, p21‐R: 5′‐CCATGAGCGCATCGCAATC‐3′, p53‐F: 5′‐CTCTCCCCCGCAAAAGAAAAA‐3′, p53‐R: 5′‐CGGAACATCTCGAAGCGTTTA‐3′, p38‐F: 5′‐TGACCCTTATGACCAGTCCTTT‐3′, p38‐R: 5′‐GTCAGGCTCTTCCACTCATCTAT‐3′, 53BP1‐F: 5′‐ATTGAACGGTTACCTCAGCCA‐3′, 53BP1‐R: 5′‐CCCAACTGTGATGAAGCAGAAT‐3′, BGal‐F: 5′‐AAAGCCTCTATCCCCTGACAT‐3′, BGal‐R: 5′‐GATCACGGACGCCATTGAAG‐3′, IL‐6‐F: 5′‐CTGCAAGAGACTTCCATCCAG‐3′, IL‐6‐R: 5′‐AGTGGTATAGACAGGTCTGTTGG‐3′, IL‐8‐F: 5′‐TCGAGACCATTTACTGCAACAG‐3′, IL‐8‐R: 5′‐CATTGCCGGTGGAAATTCCTT‐3′, IL‐1a‐F: 5′‐CGAAGACTACAGTTCTGCCATT‐3′, IL‐1a‐R: 5′‐GACGTTTCAGAGGTTCTCAGAG‐3′, Wnt5b‐F: 5′‐GGAGTACGGCTACCGCTTTG‐3′, Wnt5b‐R: 5′‐TCCTCCGATCCCTTGGCAA‐3′, Wnt5a‐F: 5′‐CTGCGGAGACAACATCGACTA‐3′, Wnt5a‐R: 5′‐CGTGGATTCGTTCCCTTTCTCTA‐3′, Prkcq‐F: 5′‐TCCGCCAGCATCCTTTGTTT‐3′, Prkcq‐R: 5′‐TCCTTGTCGAAATTGCTACAGTC‐3′, Plcb4‐F: 5′‐GGACAAGTGCTAGAATGTTCCC‐3′, Plcb4‐R: 5’‐GAAGCCGATATTCACCAGATCC‐3′, Plcb3‐F: 5′‐CGCGGGAGTAAGTTCATCAAA‐3′, Plcb3‐R: 5′‐CCTCCATGTTGGGTCCTGTC‐3′, Plcb2‐F: 5′‐AGGATAGCTGTGATGGAAGAAGG‐3′, Plcb2‐R: 5′‐GCCCAGGTGTCAGGTATGTAG‐3′, Plcb1‐F: 5′‐ACCTGAGCGGAGAAGAAAATG‐3′, Plcb1‐R: 5′‐TGTTGTGCGAGGAATTGATGAA‐3′, CaN‐F: 5′‐TGAGGATCTTTGACTCTCCTGAA‐3′, CaN‐R: 5′‐CCAGCGAAGAGCATACCATAC‐3′, Pcdha3‐F: 5′‐CACTGGCACCACTCAAGTGAA‐3′, Pcdha3‐R: 5′‐GGAGCATTTTCGGGTAATCTGAC‐3′, Nfatc4‐F: 5′‐GAGCTGGAATTTAAGCTGGTGT‐3′, Nfatc4‐R: 5′‐CATGGAGGGGTATCCTCTGAG‐3′, Nfatc3‐F: 5′‐GCTCGACTTCAAACTCGTCTT‐3′, Nfatc3‐R: 5′‐GATGTGGTAAGCCAAGGGATG‐3′, Nfatc2‐F: 5′‐TCATCCAACAACAGACTGCCC‐3′, Nfatc2‐R: 5′‐GGGAGGGAGGTCCTGAAAACT‐3′, Nfatc1‐F: 5′‐CAGTGTGACCGAAGATACCTGG‐3′, Nfatc1‐R: 5′‐TCGAGACTTGATAGGGACCCC‐3′, Myh7‐F: 5′‐CCTGCGGAAGTCTGAGAAGG‐3′, Myh7‐R: 5′‐CTCGGGACACGATCTTGGC‐3′, Gng13‐F: 5′‐AGAGCCTCAAGTACCAACTGG‐3′, Gng13‐R: 5′‐CTTCTCTACCCAAGGGTTGTTC‐3′, Fzd10‐F: 5′‐CATGCCCAACCTGATGGGTC‐3′, Fzd10‐R: 5′‐GCCACCTGAATTTGAACTGCTC‐3′, Fzd1‐F: 5′‐CAGCAGTACAACGGCGAAC‐3′, Fzd1‐R: 5′‐GTCCTCCTGATTCGTGTGGC‐3′, Cdh6‐F: 5′‐CCCTACCCAACTTTCTCAAACC‐3′, Cdh6‐R: 5′‐GAACGGCTCAGCTCATTCC‐3′, Cdh26‐F: 5′‐GCCCTTTCCTAAACTGGTTGG‐3′, Cdh26‐R: 5′‐AAGACGGTGTCACTTCTCGAT‐3′, Cdh9‐F: 5′‐GTCTGGAGTTGGTACGTCTGT‐3′, Cdh9‐R: 5′‐CCGGGTCCACTGAAAAGTATG‐3′, Camk2b‐F: 5′‐GCACGTCATTGGCGAGGAT‐3′, Camk2b‐R: 5′‐ACGGGTCTCTTCGGACTGG‐3′, Camk2a‐F: 5′‐ACCTGCACCCGATTCACAG‐3′, Camk2a‐R: 5′‐TGGCAGCATACTCCTGACCA‐3′, Pax7‐F: 5′‐TGGGGTCTTCATCAACGGTC‐3′, Pax7‐R: 5′‐ATCGGCACAGAATCTTGGAGA‐3′, 18SrRNA‐F: 5′‐AGGGGAGAGCGGGTAAGAGA‐3′, 18SrRNA‐R: 5′‐GGACAGGACTAGGCGGAACA‐3′.

### 2.8 Western blot analysis

Briefly, the total protein of each group of cells was electrophoresed on a 12% SDS‐PAGE denaturing gel, and after completion the proteins were transferred to a PVDF membrane (Millipore). After sealing and washing the membrane, incubation with primary antibodies [Rabbit anti‐Phospholipase C beta 1/PLCB1 antibody [EPR19085] (ab182359), Rabbit anti‐MYH7B antibody [EPR12290] (ab172967), Rabbit anti‐PAX7 antibody (ab187339), Rabbit anti‐CaMKII delta antibody [EPR13095] (ab181052), Rabbit anti‐NFAT1 antibody [EPR24658‐149] (ab283649), Rabbit anti‐GAPDH antibody [EPR16891] (ab181602), Abcam, MA, USA or Cell Signalling Technology, MA, USA.] was performed for 45 min at 37°C. After sufficient membrane washing, it was incubated with secondary antibodies [Goat anti‐Rabbit IgG H&L (HRP) (ab97051), Goat anti‐Mouse IgG H&L (HRP) (ab6789), Abcam, MA, USA or Cell Signalling Technology, MA, USA] for 45 min at 37°C. The membranes were then washed four times with TBST for 14 min each time at room temperature. The membranes were ultimately exposed and developed using ECL‐enhanced chemiluminescence (ECL kit, Pierce Biotechnology; Sigma‐Aldrich Chemical Co., St. Louis, MO, USA).

### 2.9 Fluorescence in situ hybridization (FISH)

The reagents and containers that we utilized were treated with DEPC water. Adherent cells were prehybridized using 10 μL/mL proteinase K (Sigma‐Aldrich Chemical), 0.2% glycine and 4% paraformaldehyde to remove proteins and again prehybridized in hybridization solution at 60°C for 2 h; the FITC‐labelled miRFISH probe (Novobiosci, Shanghai, China) was added for hybridization at 60°C overnight. After washing and dehydration, the slices were sealed and photographed.

### 2.10 Luciferase reporter assay

Our protocol conformed to previous studies.^11^, ^19^, ^21^ Briefly, the wild‐type PAX7 3′UTR fragment (5′‐ACAGAGGCTTGCCCACTGTGTCAGGCCTGGGCAGC‐3′) and the mutant PAX7 3′UTR fragment (5′‐ACAGAGGCTTATTTCCGATGTCAGGCCTGGGCAGC‐3′) were cloned into the polyclonal site of a psiCHECK‐2 luciferase reporter plasmid (Novobiosci, Shanghai, China). The psiCHECK‐PAX7‐WT‐3UTR or psiCHECK‐PAX7‐Mut‐3UTR plasmid was subsequently co‐transfected with miR‐3061–5p mimics (Novobiosci, Shanghai, China) into 293 T cells, and the luciferase activity was quantified after 48 h.

### 2.11 microRNA‐Seq

microRNA library construction and microRNA‐Seq were performed by KangChen Bio‐tech (Shanghai, China). According to their protocol, total RNA from each group of samples was extracted to prepare microRNA sequencing libraries that comprised the following steps: (1) 3′ junction ligation, (2) 5′ junction ligation, (3) cDNA synthesis, (4) PCR amplification and (5) selection of ~135–155 bp of PCR‐amplified fragments (corresponding to ~15–35 nt of small RNAs). The libraries were denatured into single‐stranded DNA molecules, captured on an Illumina flow cell, amplified in situ into clusters and sequenced for 51 cycles on an Illumina NextSeq 500 sequencer according to the vendor's instructions. For the data obtained from RNA‐Seq, the raw reads were screened using Solexa CHASTITY quality control to obtain clean reads. We obtained trimmed reads by de‐junctioning the clean reads and leaving tags ≥15 nt in length, and all trimmed reads were used to perform new microRNA sequencing using miRDeep2 software. Reads for new microRNA prediction were applied to obtain new pre‐microRNAs, and then using Novoalign software (v2.07.11) the trimmed reads were compared to the merged pre‐microRNA database (miRBase v21 pre‐microRNAs + newly predicted pre‐microRNAs), allowing up to one mismatch. Reads with a number less than 2 were discarded when calculating microRNA expression. To characterize the variation in isoforms, reads with ±4 nt in the mature body region were treated as isoforms of mature microRNAs and categorized into 5P and 3P classes according to their position in the precursor hairpin. The number of reads aligned to each mature microRNA region (±4 nt) was counted as the raw expression of the microRNA, and the samples were normalized using TPM (tag counts per million microRNA alignments). We calculated the expression levels of the mature microRNAs, the most highly expressed isoforms and all microRNA isoforms to meet the different needs of our clients. Based on the normalized tag number for all microRNA isoforms, we calculated the *p*‐value and fold‐change between the two sets of samples (with replicates), and differential microRNAs were then screened based on these values. We constructed a microRNA clustering diagram and predicted the target genes of the top 10 differential microRNAs using microRNA target gene prediction software.

### 2.12 RNA‐Seq

RNA library construction and RNA‐Seq were performed by Biomarker Technologies (BMK, Beijing, China). Briefly, total RNA was examined for purity and concentration using a NanoDrop 2000 spectrophotometer, and RNA integrity was accurately determined using an Agilent 2100/LabChip GX. Subsequently, eukaryotic mRNA was enriched with magnetic beads using oligo (dT), and mRNA was randomly interrupted by adding fragmentation buffer. The first cDNA strand and second strand were synthesized using mRNA as a template, and cDNA purification was accomplished. The purified double‐stranded cDNA was then used for end‐repair, the addition of an A‐tail and ligation of sequencing junctions. AMPure XP beads were used for fragment‐size selection, and, finally, the cDNA library was enriched using PCR. After library construction was completed, a Qubit 3.0 Fluorescence Quantification Instrument was adopted for preliminary quantification, and the concentration was ≤1 ng/μL. Subsequently, we used a Qsep400 high‐throughput analyser system to detect the insertion fragments of the library, and after the insertion fragments conformed to expectations, qPCR was used to accurately quantify the effective concentration of the library (the effective concentration of the library was >2 nM) to ensure library quality. After the library passed the quality check, PE150 mode sequencing was implemented using the Illumina NovaSeq6000 sequencing platform. After the sequencing data were downloaded, the data were analysed using the bioinformatic analysis process provided by BMKCloud (www.biocloud.net). The offline data were filtered to obtain clean data and sequence comparison with the specified reference genome, and mapped data were obtained for assessment of library quality, structure‐level analysis, differential‐expression analysis and gene‐function annotation and function enrichment.

### 2.13 H&E staining

Tissue samples were fixed in 4% paraformaldehyde, dehydrated and paraffin‐embedded. Tissue was sectioned at a 4‐μm thickness on a paraffin slicer and pasted on slides. Deparaffinization was subsequently achieved using xylene and ethanol‐gradient dehydration. We stained tissues with a haematoxylin solution at room temperature for 5 min, differentiated them in a 1% concentration of hydrochloric acid: ethanol for 30 s, added light ammonia to return the blue colour for 1 min and rinsed with distilled water for 5 min. We subsequently stained sections with an eosin solution at room temperature for 2 min and rinsed them with distilled water for 2 min; slides then underwent ethanol‐gradient destaining. Sections were permeabilized with xylene for 2 min and sealed with neutral gum.

### 2.14 Immunofluorescence (IF) staining

Briefly, all fresh tissues were fixed by immersion in 4% paraformaldehyde (Sigma‐Aldrich, St. Louis, MO, USA) for 30 min at room temperature, and we performed ethanol‐gradient dehydration, paraffin embedding, sectioning (at a 6‐μm thickness) and deparaffinization by invasion with xylene. Tissue sections were blocked with immunohistochemical blocking solution (Beyotime Biotechnology Co., Ltd., Zhejiang, China) at 37°C for 30 min, the sealing solution was discarded and the sections were washed three times with immunohistochemical washing solution (Beyotime Biotechnology Co., Ltd., Zhejiang, China) for 5 min each at room temperature. Then, primary antibodies [Mouse anti‐AMH antibody [5/6] (ab24542), Rabbit anti‐Ki‐67 (D3B5) antibody (#9129), Rabbit anti‐phospho‐histone H2A.X (Ser139) antibody (#2577), Rabbit anti‐phospholipase C beta 1/PLCB1 antibody [EPR19085] (ab182359), Rabbit anti‐MYH7B antibody [EPR12290] (ab172967), Rabbit anti‐PAX7 antibody (ab187339), Rabbit anti‐CaMKII delta antibody [EPR13095] (ab181052), Rabbit anti‐NFAT1 antibody [EPR24658‐149] (ab283649), Rabbit anti‐GAPDH antibody [EPR16891] (ab181602), Abcam, MA, USA or Cell Signalling Technology, MA, USA.] were added and incubated at 37°C for 45 min. The antibodies were discarded and slides were washed three times with an immunohistochemical wash solution (Beyotime Biotechnology Co., Ltd., Zhejiang, China) for 5 min each at room temperature. Then, secondary antibodies [goat anti‐rabbit IgG H&L (Alexa Fluor® 555) (ab150078), Goat Anti‐Mouse IgG H&L (Alexa Fluor® 555) (ab150114), Goat Anti‐Mouse IgG H&L (Alexa Fluor® 647) (ab150115), Goat Anti‐Rabbit IgG H&L (Alexa Fluor® 647) (ab150079), Abcam, MA, USA] were added, and slides were incubated at 37°C for 45 min. The antibodies were discarded, and an immunohistochemical washing solution (Beyotime Biotechnology Co., Ltd., Zhejiang, China) was added to immerse the sections three times for 5 min each at room temperature. Finally, the slices were blocked by adding an immunofluorescence blocking solution (Sigma‐Aldrich, St. Louis, MO, USA).

### 2.15 Isolation and enrichment of exosomes of follicular fluid origin

Follicular fluid was collected from 10 POF patients and 10 healthy women, centrifuged at 800 × *g* for 5 min to remove cellular precipitates, centrifuged at 10,000 × *g* for 30 min to remove cellular debris and centrifuged at 100,000 × *g* in an ultracentrifuge for 1.5 h. The supernatant was discarded; the precipitates were resuspended in a 2.5‐mol/L sucrose solution; and suspensions were spiked into the upper layers of 2.25, 2.0, 1.75, 1.5, 1.25, 1.0, 0.75, 0.5 and 0.25 mL of a linear iodixanol gradient and underwent 100,000 × *g* ultracentrifugation for 2 h. We collected the layer containing 1.0 mL sucrose in PBS buffer dilution, centrifuged at 100,000 × *g* for 1.5 h and resuspended the precipitate with the PBS buffer containing protease inhibitors for further analysis. Informed consent for the human samples was obtained from all patients according to consent regulation #2022–21 and approved by the Ethics Review Committee of Henan Province Hospital of Traditional Chinese Medicine as authorized by the Ministry of Health and the Municipal Government. Our study was conducted following the Declaration of Helsinki.

### 2.16 Statistical methods

The data generated by this experiment were expressed as mean ± standard deviation (mean ± SD) and analysed with analysis of variance or independent‐sample *t* tests using SPSS 10.0 statistical software. Differences were considered to be statistically significant at *p* < 0.05.

## 3 RESULTS

### 3.1 miR‐3061 is highly expressed in OGCs of the POF mouse model

**FIGURE 1:**
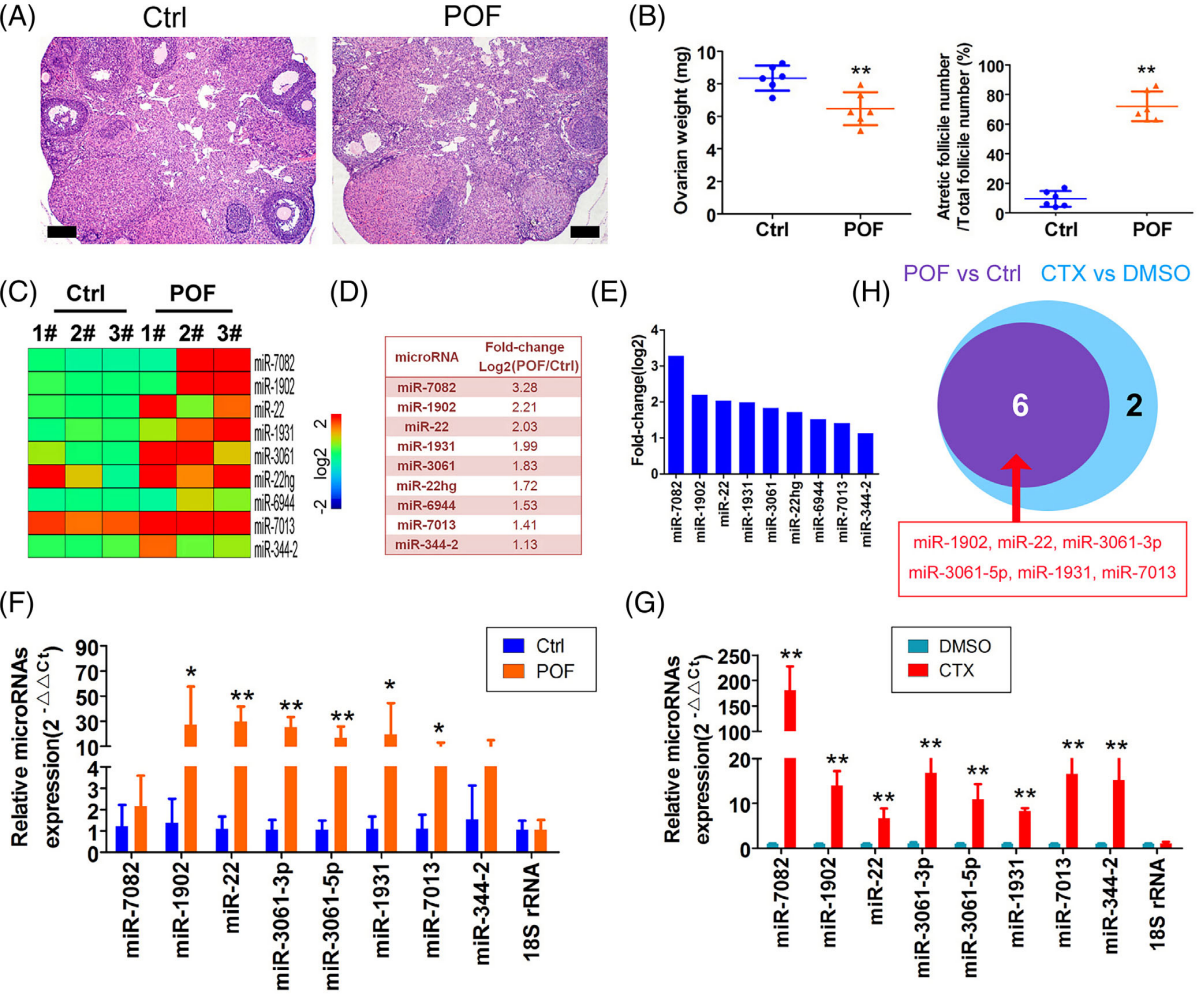
microRNA‐3061 is highly expressed in ovarian granulosa cells in an ex vivo model of POF. (A) H&E pathologic staining staining revealed that the POF ovary was significantly atrophied and the numbers of follicles at all levels were reduced.; magnification, 200×; Scale bar = 30 μm. (B) The weight of the POF ovary was lower than that of the control group, and the proportion of atretic follicles of POF group was augmented compared with that of the control group. **p < 0.01 vs. Ctrl; *t* test; *n* = 6. (C) Heatmap depicting microRNA‐Seq assay indicated that there were nine microRNAs with elevated expression levels in the granulosa cells of the ovaries of mice in the POF group. (D) Summary of significantly expressed microRNAs. (E) Statistical results of differential expression of microRNAs between the model and control. (F) The qPCR result showed that the expression levels of six microRNAs were significantly elevated in the ovarian tissues of POF mice relative to the control group. *p < 0.05 vs. Ctrl; **p < 0.01 vs. Ctrl; *t* test; *n* = 6. (G) The qPCR result showed that the expression levels of eight microRNAs were raised in the CTX‐treated mOGC group compared with the control group. *p < 0.05 vs. Ctrl; **p < 0.01 vs. Ctrl; *t* test; *n* = 6. (H) Results of the intersection comparison of microRNA expression levels in the in vivo and ex vivo POF models.

To study the differential microRNA expression profile during POF development, we constructed a CTX‐induced POF mouse model. The results of H&E staining revealed that the ovarian tissue of mice in the POF group was significantly atrophied, the numbers of follicles at all levels were reduced, the weight of the ovary was lower than that of the control group and the proportion of atretic follicles was augmented compared with that of the Ctrl group (Figure 1A,B). Subsequently, the results of high‐throughput sequencing of the microRNA transcriptome indicated that there were nine microRNAs with elevated expression levels (log2[POF/Ctrl] > 1) in the granulosa cells of the ovaries of mice in the POF group (miR‐7082, miR‐1902, miR‐22, miR‐1931, miR‐3061, miR‐22hg, miR‐6944, miR‐7031 and miR‐34‐2) (Figure 1C–E). qPCR showed that the expression levels of microRNAs such as miR‐1902, miR‐22, miR‐3061‐3p, miR‐3061–5p, miR‐1931 and miR‐7031 were significantly elevated in the ovarian tissues of POF mice relative to the control group (Figure 1F). In addition, qPCR demonstrated that the expression levels of microRNAs such as miR‐7082, miR‐1902, miR‐22, miR‐3061‐3p, miR‐3061–5p, miR‐1931, miR‐7031 and miR‐344‐2 were raised in the CTX‐treated mOGC group compared with the control group (Figure 1G). By determining their intersection, we ultimately identified six microRNAs (miR‐1902, miR‐22, miR‐3061‐3p, miR‐3061–5p, miR‐1931 and miR‐7031) as significantly elevated in their expression in the ex vivo POF model (Figure 1H). These experimental results suggested that miR‐3061 was highly expressed in the OGCs of our POF mouse model.

### 3.2 The mOGCs specific knock‐in of miR‐3061 induces the emergence of the pathological phenotype of POF in mice

**FIGURE 2:**
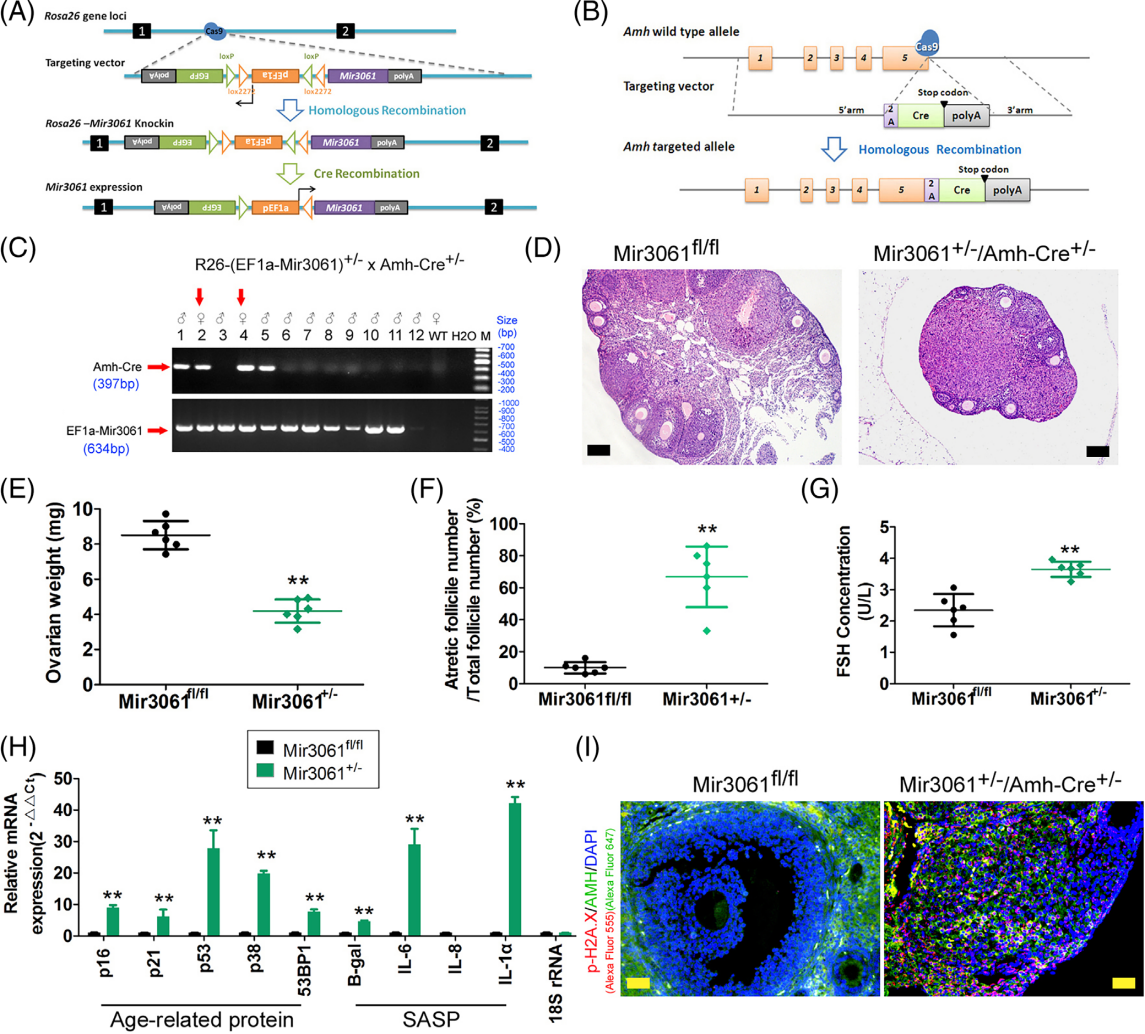
miR‐3061^+/−^/AMH‐Cre^+/−^ transgenic mice exhibit a POF pathologic phenotype. (A) Construction strategy for miR‐3061^+/−^ knock‐in mice. (B) Construction strategy for AMH‐Cre^+/−^ fusion protein transgenic mice. (C) PCR characterization of miR‐3061^+/−^/AMH‐Cre^+/−^ positive transgenic mice. (D) H&E pathologic staining results revealed that miR‐3061^+/−^/AMH‐Cre^+/−^ mice manifested significantly pathological phenotype of POF; magnification, 200×; Scale bar = 30 μm. (E) Ovarian weight assay results revealed that miR‐3061^+/−^/AMH‐Cre^+/−^ mice manifested significantly reduction in ovarian weight. **p < 0.01 vs. miR‐3061^fl/fl^; *t* test; *n* = 6. (F) miR‐3061^+/−^/AMH‐Cre^+/−^ mice manifested significantly elevated in percentage of atretic follicles. **p < 0.01 vs. miR‐3061^fl/fl^; *t* test; *n* = 6. (G) ELISA results indicated that peripheral blood FSH concentrations in miR‐3061^+/−^/AMH‐Cre^+/−^ mice were significantly augmented relative to those in the control group. **p < 0.01 vs. miR‐3061^fl/fl^; *t* test; *n* = 6. (H) The qPCR assay result showed that the expression levels of the pro‐senescence and SASP genes in OGCs of miR‐3061^+/−^/AMH‐Cre^+/−^ transgenic mice were elevated relative to control mice. **p < 0.01 vs. miR‐3061^fl/fl;^ *t* test; *n* = 6. (I) IF staining results showed high expression of cellular aging and the DNA damage‐related protein p‐H2A.X in OGCs (AMH+) of miR‐3061^+/−^/AMH‐Cre^+/−^ mice; magnification, 200×; Scale bar = 30 μm.

We successfully constructed miR‐3061^+/−^ heterozygous knock‐in (knock‐in) mice and AMH‐Cre^+/−^ fusion gene heterozygous‐expression mice using CRISPR/Cas9 gene‐editing technology. By crossing the two mouse strains, we obtained miR‐3061^+/−^/AMH‐Cre^+/−^ double‐transgenic mice that specifically expressed exogenous knock‐in miR‐3061 molecules in OGCs (AMH+) (Figure 2A–C). Pathological tests revealed that miR‐3061^fl/fl^ (control) mice depicted numerous follicles at all stages that were visible in the ovarian tissue, almost no atretic follicles and a loose ovarian structure; whereas miR‐3061^+/−^/AMH‐Cre^+/−^ mice manifested significantly reduced and shrunken ovarian tissue, occasional healthy follicles, a large number of atretic follicles and tight and homogenous ovarian structure—the latter reflecting the pathological phenotype of POF (Figure 2D–F). ELISA results indicated that peripheral blood FSH concentrations in miR‐3061^+/−^/AMH‐Cre^+/−^ mice were significantly augmented relative to those in the control group (Figure 2G). We subsequently discerned via qPCR that the expression levels of the pro‐senescence genes p16, p21, p53, p38 and 53BP1 and SASP genes B‐gal, IL‐6, IL‐8 and IL‐1a in OGCs of miR‐3061^+/−^/AMH‐Cre^+/−^ transgenic mice were elevated relative to control mice (Figure 2H). Finally, IF staining also showed high expression of cellular aging and the DNA damage‐related protein p‐H2A.X in OGCs (AMH+) of miR‐3061^+/−^/AMH‐Cre^+/−^ mice (Figure 2I). These results suggested that miR‐3061^+/−^/AMH‐Cre^+/−^ mOGCs showed pathological aging and the POF pathological phenotype.

### 3.3 Transcription factor PAX7 is a potential target gene of miR‐3061

**FIGURE 3:**
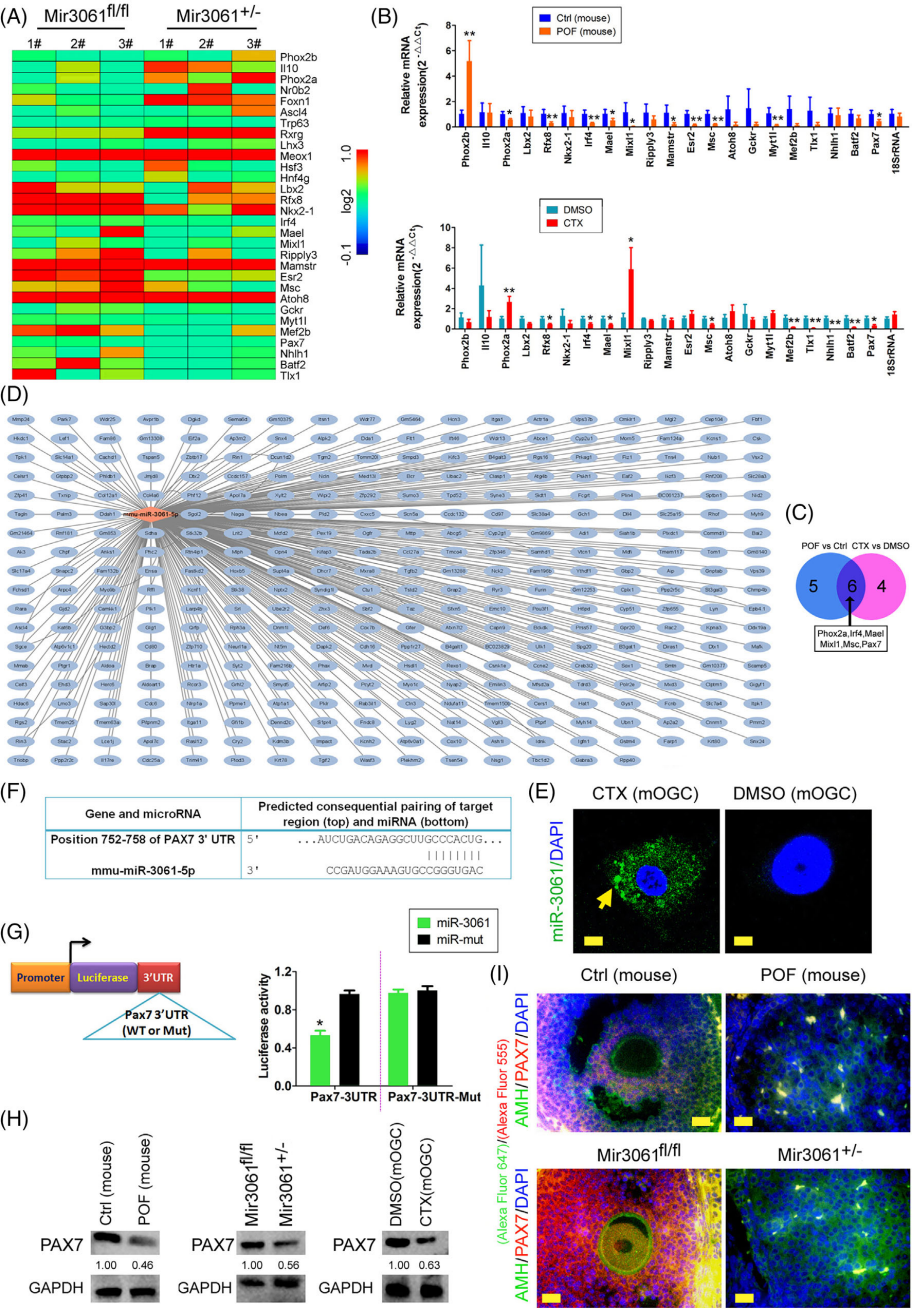
microRNA‐3061 negatively regulates the expression of the PAX gene. (A) RNA‐Seq heatmap results suggest significant differential expression of transcription factors. (B) The qPCR results then indicated that a total of 11 transcription factors were expressed at significantly higher levels in the ovarian tissues of POF mice than in the control group. *p < 0.05 vs. Ctrl; **p < 0.01 vs. Ctrl; *t* test; *n* = 6. (C) Results of intersection comparison of transcription factors expression levels indicated that six transcription factors were significantly downregulated expression in ex vivo and in vivo POF models. (D) microRNA‐3061 target gene prediction results. (E) FISH results showed a high hybridization signal for microRNA‐3061–5p in the group of CTX‐treated mOGCs; magnification, 630×; Scale bar = 100 μm. (F) Prediction results showed that an eight‐nucleotide sequence of a 752–758 bp nucleic acid in the 3′UTR of the PAX7 gene that was fully complementary to miR‐3061–5p. (G) Luciferase reporter assay results suggested that PAX7 was a target gene negatively regulated by microRNA‐3061. *p < 0.05 vs. Ctrl; *t* test; *n* = 3. (H) Western blot assay results indicated that PAX7 protein expression levels as significantly lower than those in the control group, both in POF mouse and in miR‐3061^+/−^/AMH‐Cre^+/−^ mice, as well as in the CTX‐treated mOGC group. (I) IF staining results indicated that PAX7 protein expression levels as significantly lower than those in the control group, both in POF mouse, and in miR‐3061^+/−^/AMH‐Cre^+/−^ mice; magnification, 400×; Scale bar = 60 μm.

We exploited RNA‐Seq high‐throughput transcriptomic sequencing to characterize the variability of transcription factor expression profiles between miR‐3061^+/−^/AMH‐Cre^+/−^ mice and control mOGCs (Table S1 and Figure 3A) and identified a total of 1021 transcription factors, of which 33 were statistically different (log2[miR‐3061^+/−^/AMH‐Cre^+/−^ vs. miR‐3061^fl/fl^ ≥1.5 or ≤ −1.5]). qPCR results then indicated that a total of 11 transcription factors were expressed at significantly higher levels in the ovarian tissues of POF mice than in the control group (Figure 3B) and that these same transcription factors were markedly expressed in the CTX‐treated mOGC group compared with the control group (Figure 3B). We noted that by describing their intersection, six transcription factors (Phox2a, Irf4, Mael, Mixl1, Msc and Pax7) were ultimately identified as showing significantly downregulated expression in ex vivo and in vivo POF models (Figure 3C). To ascertain the target genes negatively regulated by microRNA‐3061, we applied the bioinformatics tool TargetScan for the prediction of candidate target genes. The prediction results suggested that more than 340 candidate genes were targeted by microRNA‐3061 (microRNA‐3061–5p) (Figure 3D) and that the transcription factor PAX7 was among them. In addition, using FISH we identified a high hybridization signal for microRNA‐3061–5p in the group of CTX‐treated mOGCs (Figure 3E). A comparison of gene sequences revealed the presence of an eight‐nucleotide sequence of a 752–758 bp nucleic acid in the 3′UTR of the PAX7 gene that was fully complementary to miR‐3061–5p (Figure 3F). To verify the negative regulatory effect of microRNA‐3061–5p on PAX7, we constructed a luciferase reporter plasmid, and our assay results showed that when microRNA‐3061–5p was overexpressed in the cells, the luciferase reporter enzyme carrying the WT PAX7 3′ UTR activity was significantly diminished, while none of the remaining combinations affected the expression activity of the enzyme (Figure 3G). Finally, the results of western blot assay depicted PAX7 protein expression levels as significantly lower than those in the healthy group, both in OGCs of the POF mouse model and in OGCs of miR‐3061^+/−^/AMH‐Cre^+/−^ mice, as well as in the CTX‐treated mOGC group (Figure 3H). The results of the IF experiment were also consistent with the results of western blot analysis (Figure 3I). These results therefore suggested that the transcription factor PAX7 was one of the potential negative regulatory target genes of miR‐3061.

### 3.4 miR‐3061 inhibits the expression of Wnt (Wnt/Ca^2+^) signalling pathway genes by negatively regulating PAX7 expression

**FIGURE 4:**
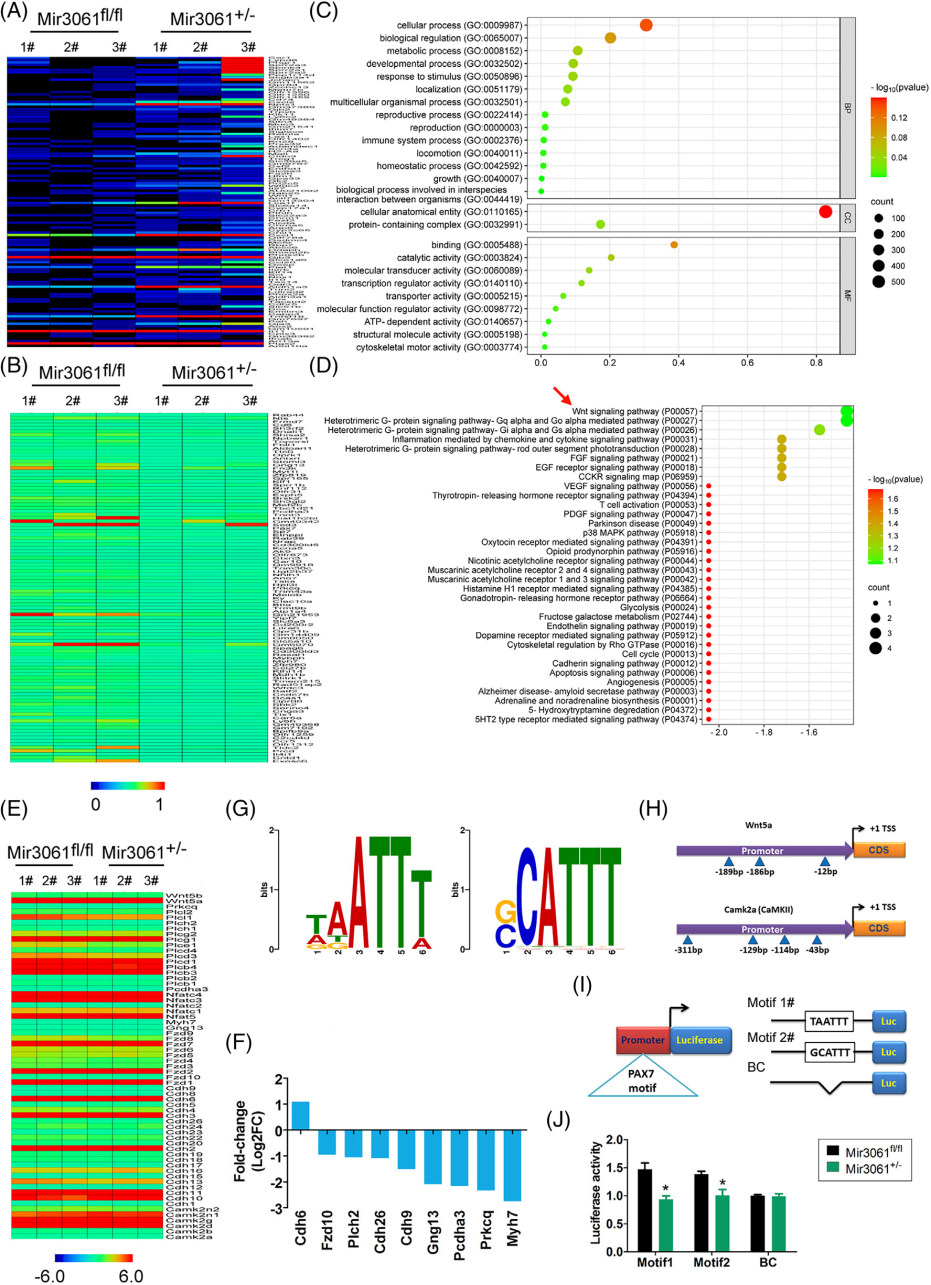
PAX7 regulates the expression of Wnt/Ca^2+^ signalling pathway genes. (A) RNA‐Seq heatmap results for the set of significantly upregulated 118 DEGs. (B) RNA‐Seq heatmap results for the set of significantly downregulated 109 DEGs. (C) Prediction results of GO analysis for the set of significantly downregulated DEGs were principally concentrated in cellular process (BP), cellular anatomical entity (CC) and transcription regulator activity (MF). (D) Prediction results of PATHWAY analysis for set of significantly downregulated DEGs were primarily involved in signalling pathways such as the Wnt (Wnt/Ca^2+^) signalling pathway and heterotrimeric G‐protein signalling pathway. (E) RNA‐Seq results of all node proteins of the Wnt/Ca^2+^ signalling pathway. (F) Statistical analysis of significant differential expression of key genes of the Wnt/Ca^2+^ signalling pathway. (G) Recognition by the transcription factor PAX of the cis‐acting site motif on gene promoters. (H) Prediction results of the PAX binding site motif on the promoter region of the Wnt5a and Camk2a genes. (I) Schematic representation of the insertion sequence and structure of the luciferase reporter plasmid DNA. (J) Luciferase reporter assay results indicated that PAX7 enhanced the activity of luciferase containing the bound motif. *p < 0.05 vs. blank control (BC); *t* test; *n* = 3.

To determine the overall effects of microRNA‐3061 on gene expression levels in OGCs after specific knock‐in of microRNA‐3061 in our cells, we implemented RNA‐Seq high‐throughput transcriptomic sequencing analysis. RNA‐Seq detected a total of 24,971 gene transcripts, of which 118 genes showed significantly upregulated expression (log2[miR‐3061 ^+/−^/AMH‐Cre^+/−^ vs. miR‐3061^fl/fl^] > 2) (Figure 4A;, Table S1) while 109 genes were significantly downregulated (log2[miR‐3061^+/−^/AMH‐Cre^+/−^ vs. miR‐3061^fl/fl^] < −2) (Figure 4B; Table S1). Considering the negative regulatory effects of microRNAs on target genes, we analysed the functions of the transcripts of the downregulated genes and the signalling pathways to which they belonged. The results of GO analysis showed that significantly downregulated genes were principally concentrated in cellular process (BP) in miR‐3061^+/−^/AMH‐Cre^+/−^ mOGCs, cellular anatomical entity (CC), transcription regulator activity (MF) and other functional distribution groups (Figure 4C). KEGG prediction showed that in miR‐3061^+/−^/AMH‐Cre^+/−^ mOGCs, the significantly downregulated genes were primarily involved in signalling pathways such as the Wnt (Wnt/Ca^2+^) signalling pathway and heterotrimeric G‐protein signalling pathway (Figure 4D). Since the Wnt (Wnt/Ca^2+^) signalling pathway was ranked first, we analysed the gene expression levels of the key nodes in this signalling pathway, and the results showed a tendency for downregulation of the genes in this signalling pathway (Figure 4E,F). Bioinformatics software prediction revealed that the promoters of two key genes in the Wnt (Wnt/Ca^2+^) signalling pathway, Wan5a and Camk2a, contained multiple cis‐binding sites for the PAX7 motif (Figure 4G,H). Subsequently, we constructed a luciferase reporter assay plasmid DNA system (Figure 4I) and showed that the reporter with the WT PAX7 cis‐binding site motif inserted at the promoter site plasmid DNA exhibited high luciferase activity in miR‐3061^+/−^/AMH‐Cre^+/−^ mOGCs, whereas all of the above plasmids showed significantly reduced luciferase activity in miR‐3061^fl/fl^ mOGCs (Figure 4J).

**FIGURE 5:**
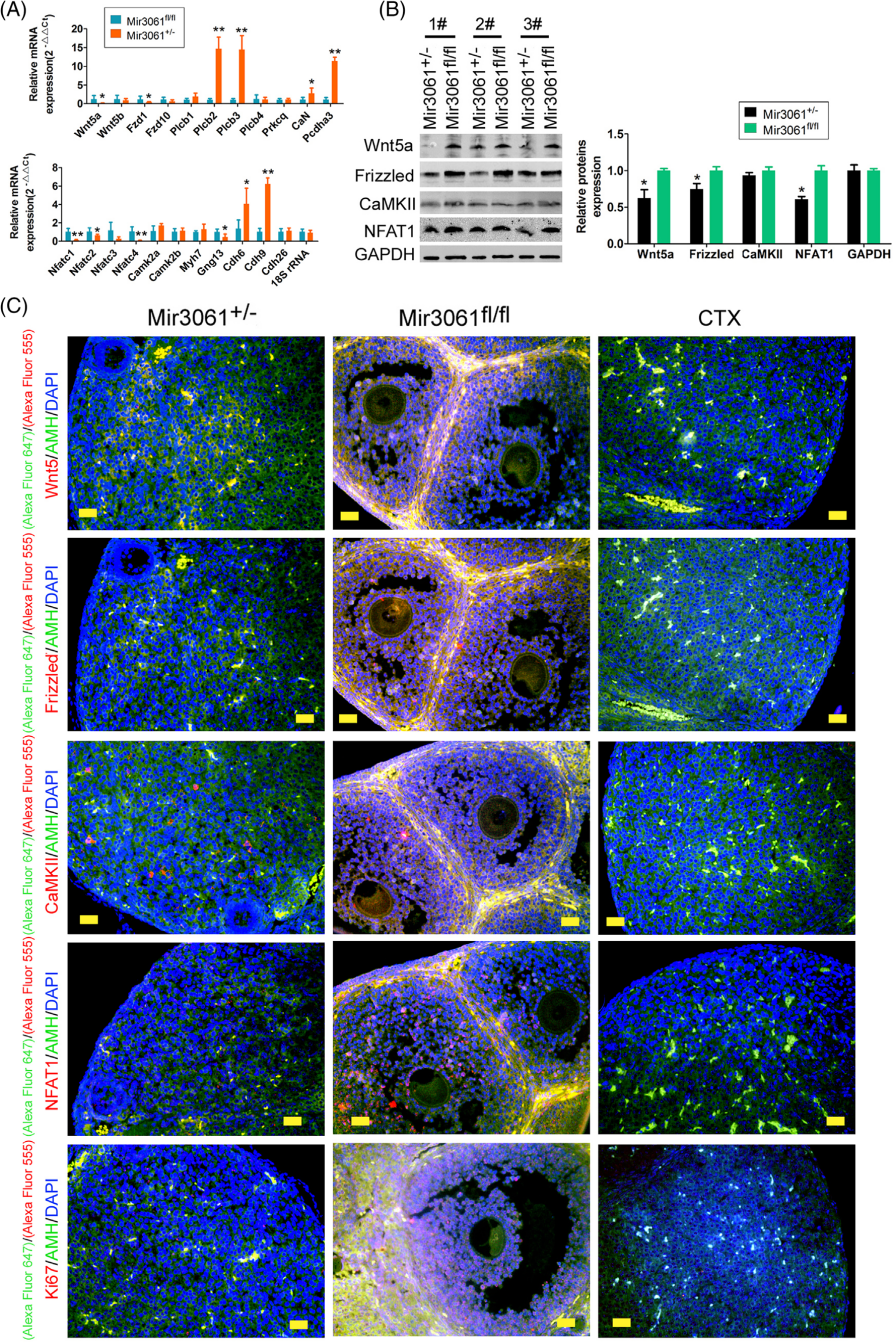
microRNA‐3061 downregulates the expression of Wnt/Ca^2+^ signalling pathway genes in OGCs. (A) qPCR assay results indicated that the expression levels of key genes of the Wnt/Ca^2+^ signalling pathway were significantly reduced in OGCs of miR‐3061^+/−^/AMH‐Cre^+/−^ transgenic mice. *p < 0.05 vs. miR‐3061^fl/fl^; **p < 0.01 vs. miR‐3061^fl/fl^; *t* test; *n* = 6. (B) Western blot assay results indicated that the expression levels of key proteins of the Wnt/Ca^2+^ signalling pathway were significantly reduced in OGCs of miR‐3061^+/−^/AMH‐Cre^+/−^ transgenic mice. *p < 0.05 vs. miR‐3061^fl/fl^; **p < 0.01 vs. miR‐3061^fl/fl^; *t* test; *n* = 6. (C) IF staining results showed that the expression levels of Wnt5, Frizzled, CaMKII, NFAT1 and Ki67 proteins were significantly reduced in miR‐3061^+/−^/AMH‐Cre^+/−^ mOGCs in mOGCs (AMH+); magnification, 400×; Scale bar = 60 μm.

We also identified the expression levels of Wnt/Ca^2+^ signalling pathway genes using qPCR, determining that the expression levels of Wat5a, Fzd1, Nfatc1, Nfatc2, Nfatc4, Gng13 and other genes were significantly lower in miR‐3061^+/−^/AMH‐Cre^+/−^ mOGCs than in the miR‐3061^fl/fl^ group (Figure 5A). Western blot analysis indicated attenuated expression levels of proteins such as Wnt5, Frizzled and NFAT1 in miR‐3061^+/−^/AMH‐Cre^+/−^ mOGCs relative to the control group (Figure 5B). IF assay also showed that the expression levels of proteins such as Wnt5, Frizzled, CaMKII, NFAT1 and Ki67 were significantly reduced in miR‐3061^+/−^/AMH‐Cre^+/−^ mOGCs in mOGCs (AMH+) (Figure 5C). Therefore, the above experimental results suggested that miR‐3061 was likely to inhibit the expression of Wnt (Wnt/Ca^2+^) signalling pathway genes and proteins by negatively regulating the expression of the trans‐regulatory element PAX7.

### 3.5 Overexpression of miR‐3061 leads to inhibition of cellular proliferation and downregulation of gene expression in the Wnt/Ca^2+^ signalling pathway in mOGCs

**FIGURE 6:**
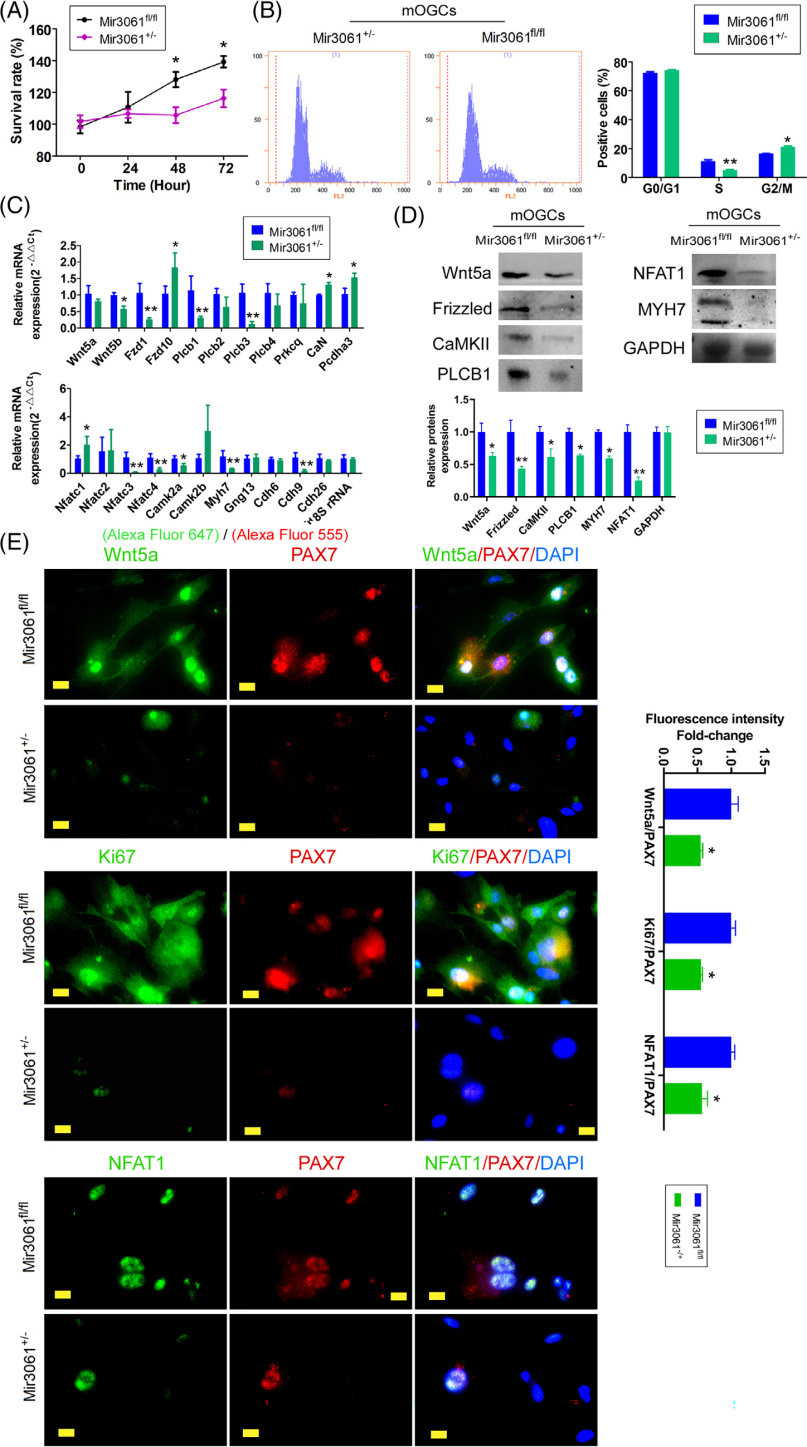
microRNA‐3061 inhibits proliferation and cell‐cycle progression of mOGCs. (A) CCK8 assay results indicated that the in vitro proliferation rate of OGCs in miR‐3061^+/−^/AMH‐Cre^+/−^ transgenic mice was significantly lower than that of controls. *p < 0.05 vs, miR‐3061^fl/fl^; *t* test; *n* = 3. (B) FCM results indicated that OGCs from miR‐3061^+/−^/AMH‐Cre^+/−^ transgenic mice exhibited a significantly reduced time in the S‐phase and significantly greater time in the G2/M phase. *p < 0.05 vs. miR‐3061^fl/fl^; **p < 0.01 vs. miR‐3061^fl/fl^; *t* test; *n* = 6. (C) The qPCR results indicated that overexpression of miR‐3061 significantly reduced the expression of key genes of the Wnt/Ca^2+^ signalling pathway in OGCs from transgenic mice. *p < 0.05 vs. miR‐3061^fl/fl^; **p < 0.01 vs. miR‐3061^fl/fl^; *t* test; *n* = 3. (D) Western blot results indicated that overexpression of miR‐3061 significantly reduced the expression of key proteins of the Wnt/Ca^2+^ signalling pathway in OGCs from transgenic mice. *p < 0.05 vs. miR‐3061^fl/fl^; **p < 0.01 vs. miR‐3061^fl/fl^; *t* test; *n* = 3. (E) IF staining results showed that showed that the expression levels of Wnt5, NFAT1, Ki67 and PAX7 proteins were lower in OGCs from miR‐3061^+/−^/AMH‐Cre^+/−^ transgenic mice than in the control group; magnification, 400×; Scale bar = 60 μm. *p < 0.05 vs. miR‐3061^fl/fl^; **p < 0.01 vs. miR‐3061^fl/fl^; *t* test; *n* = 3.

To determine the effects of miR‐3061 overexpression on the physiological and biochemical functions of mOGCs, the miR‐306^+/−^/AMH‐Cre^+/−^ and miR‐3061^fl/fl^ mOGCs were isolated and cultured in vitro. CCK‐8 assay results indicated that the proliferation rate of miR‐3061^+/−^ mOGCs in vitro was significantly lower than that of control cells (Figure 6A). FCM results showed that the proportion of cells in the S‐phase for miR‐3061^+/−^ mOGCs was significantly lower than that of control cells and that the proportion in the G2/M phase cells was significantly higher, suggesting that a majority of the miR‐3061^+/−^ mOGCs were in the G2/M phase of the cell cycle (Figure 6B). In addition, the results of our qPCR assay showed that the expression levels of several key genes of the Wnt (Wnt/Ca^2+^) signalling pathway in miR‐3061^+/−^ mOGCs were significantly reduced compared with those of miR‐3061^fl/fl^ mOGCs in the control group (Figure 6C). The results of our western blot analysis also showed that the expression levels of proteins such as Wnt5, Frizzled, CaMKII, NFAT1, PLCN1 and MYH7 were significantly lower in miR‐3061^+/−^ mOGCs than in the control group (Figure 6D). The results of our IF assay showed that the expression levels of Wnt5, NFAT1, Ki67 and PAX7 proteins were lower in miR‐3061^+/−^ mOGCs than in the control group (Figure 6E). Thus, these experimental data suggested that overexpression of miR‐3061 led to the inhibition of cellular proliferation and downregulation of the expression of Wnt/Ca^2+^ signalling pathway genes in mOGCs.

### 3.6 Expression levels of mRNAs for key factors of the Wnt/Ca^2+^ signalling pathway in human follicular fluid‐derived exosomes correlate with hormonal levels in patients with POF

**FIGURE 7:**
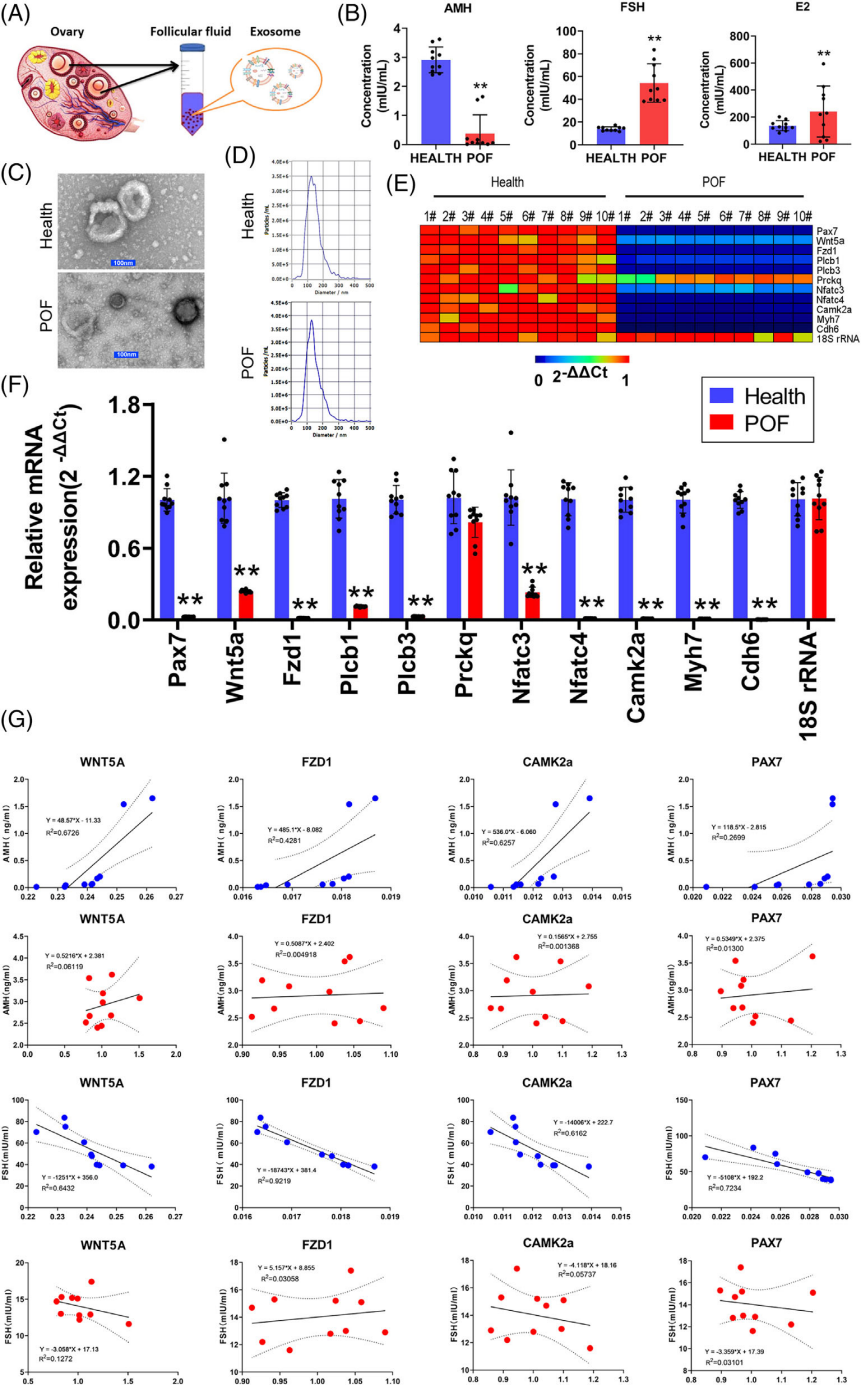
Correlation analysis between mRNA expression of key genes of the Wnt/Ca^2+^ signalling pathway and hormones in follicular fluid‐derived exosomes. (A) Collection of exosomes from follicular fluid sources in women. (B) Differences in peripheral blood AMH, E2 and FSH hormone levels between groups. **p < 0.01 vs. healthy group; *t* test; *n* = 10. (C) Morphologic identification via TEM of follicular fluid‐derived exosomes (scale bar = 100 nm). (D) The results of particle sizing analysis revealed that the FF‐exosomes diameters ranged from 50 to 200 nm. (E) Heatmap of qPCR assay results for mRNA expression levels of key genes of the Wnt/Ca^2+^ signalling pathway in follicular fluid‐derived exosomes. (F) qPCR results indicated that the expression levels of key genes of the Wnt/Ca^2+^ signalling pathway were significantly lower in the follicular fluid‐derived exosomes of POF patients relative to the control group. **p < 0.01 vs. healthy group; *t* test; *n* = 10. (G) Correlation analysis between mRNA expression levels of key genes of the Wnt/Ca^2+^ signalling pathway and reproductive hormone levels in peripheral blood.

To determine whether there was a correlation between the mRNA expression levels of key factors of the Wnt/Ca^2+^ signalling pathway in follicular fluid‐derived exosomes (FF‐exosomes) from POF patients and sex hormone levels, we established a small sample cohort of 40 individuals (20 healthy individuals and 20 POF patients) and used a gradient‐density ultracentrifugation method to enrich their FF‐exosomes (Figure 7A). We found that the levels of AMH were significantly lower in the peripheral blood of POF patients than in the healthy population. However, the levels of FSH and E2 were significantly higher in the serum of POF patients than in the healthy population (Figure 7B). The results of particle sizing and transmission electron microscopy suggested that the FF‐exosomes portrayed a typical microvesicular structure and that their diameters ranged from 50 nm to 200 nm (Figure 7C,D). qPCR assay results indicated that the vast majority of the key genes in the Wnt/Ca^2+^ signalling pathway were expressed at significantly lower levels in POF FF‐exosomes than in healthy FF‐exosomes (Figure 7E,F). Finally, the results of correlation analysis indicated that the expression levels of Wnt5a, Fzd1, Camk2a and Pax7 mRNAs both in POF‐FF‐exosomes and healthy FF‐exosomes were positively correlated with the concentrations of AMH in patient peripheral blood; while the expression levels of Wnt5a, Camk2a and Pax7 mRNAs both in POF FF‐exosomes and healthy FF‐exosomes were negatively correlated with the levels of E2 in peripheral blood (Figure 7G). Thus, these findings suggested that the expression levels of Wnt5a, Fzd1, Camk2a and Pax7 genes in human follicular fluid‐derived exosomes exhibited a significant correlation with hormonal levels in POF patients and constituted a potential biodiagnostic marker.

## 4 DISCUSSION

The ovary enacts two major functions: one is to facilitate oocyte development, maturation and completion of ovulation; and the other is the biosynthesis of steroid hormones.^22^ Steroid hormones are essential for the development of pubertal secondary sex characteristics, subsequent ovarian function, oocyte maturation and the establishment and maintenance of pregnancy.^22^ The WNT/β‐catenin pathway—now recognized as an important contributor to the regulation of ovarian steroidogenesis—may be one of the key signalling pathways involved in gonadal hormone regulation.^22^ Several proteins of the Wnt signalling pathway have been reported to be stably expressed in OGCs and thereby regulate follicular development, ovulation and luteinization.^23^ Chemotherapeutic agents such as doxorubicin, paclitaxel and cisplatin inhibit the expression of Wnt3 and β‐catenin proteins in human ovarian luteinized granulosa cells to varying degrees.^23^ Furthermore, Chen et al. found that puerarin alleviated oxidative stress damage and improved the survival rate of female germline stem cells by promoting the expression of factors belonging to the Wnt/β‐catenin signalling pathway, thereby improving the symptoms of POF.^23^ El‐Derany et al. also reported that bone marrow mesenchymal stem cells maintained the physiological activity needed for follicular proliferation and differentiation and improved the symptoms of radiation‐induced POF by maintaining the stable expression of TGF‐β, Wnt/β‐catenin and Hippo signalling pathways.^24^ Some of the aforementioned studies revealed that the expression of Wnt signalling pathway genes exerted a positive effect on maintaining ovarian function and improving POF symptoms. However, extant studies only described changes in the expression levels of Wnt signalling pathway factors in the pathophysiological state of POF, while no detailed studies have been reported on the causes or mechanisms underlying the differential expression of Wnt signalling pathway genes in POF development.

In the present study, we identified significantly downregulated expression of the Wnt signalling pathway in mOGCs of our POF model using high‐throughput transcriptomic sequencing, and this was consistent with existing reports and further confirmed the importance of the Wnt pathway in the maintenance of ovarian function. We further demonstrated that the expression levels of Wnt/Ca^2+^ signalling pathway factors—but not those of the Wnt/β‐catenin signalling pathway—were significantly downregulated in mOGCs from miR‐3061 conditional knock‐in mice, constituting an unprecedented pathway belonging to the non‐canonical Wnt signalling pathway.^25^, ^26^, ^27^, ^28^, ^29^ Considering the complexity of the Wnt signalling pathway and the involvement of multiple downstream protein nodes, it is reasonable to hypothesize that the regulatory mechanisms within the Wnt signalling pathway as modulated by POF induced by different factors are somewhat disparate. The first and most significant feature of this study, then, was the provision of an in‐depth analysis of the molecular biological mechanism subserving the downregulation of Wnt/Ca^2+^ signalling pathway expression in mOGCs from our POF model. We confirmed that the transcriptional expression of the key factors of the Wnt/Ca^2+^ signalling pathway was regulated by the transcription factor PAX7 through a variety of molecular biology experiments. In turn, PAX7 was one of the negatively regulated target genes of miR‐3061–5p. We demonstrated that POF caused by chemotherapeutic agents induced significantly elevated expression of miR‐3061 (miR‐3061–5p/3p) in mOGCs by comparing both ex vivo and in vivo models of CTX‐induced POF and that the upregulated expression of miR‐3061 downregulated the expression of Wnt/Ca^2+^ signalling pathway components by inducing PAX7 RNAi. Thus, it can be seen that the mechanisms governing POF are very complex and involve a regulatory cascade of multiple genes. The regulation between genes also spans the spectrum from ordinary trans‐acting elements that regulate cis‐acting sites (i.e., transcriptional‐level regulation) to non‐coding RNA‐induced RNAi (i.e., epigenetic regulation). Although most previous studies have focused on the Wnt/β‐catenin signalling pathway, our study showed that the onset of POF was likely to be closely related to the Wnt/Ca^2+^ signalling pathway; this finding provides a novel perspective and drug target for future drug development.

The second highlight of this study was the validation of the correlation between the mRNA expression levels of key genes of the Wnt/Ca^2+^ signalling pathway and sex hormone levels in follicular fluid‐derived exosomes from POF patients. It is generally accepted from a growing number of studies that bodily fluid‐derived cellular exosomes can serve as a potential noninvasive diagnostic marker for POF. However, there have been several reports showing that significant differences in mRNAs, steroid hormones, proteins and non‐coding RNAs contained in follicular fluid exosomes of patients with ovarian insufficiency were found to exist between POF patients and healthy subjects and that there was a correlation with the severity of the disease. However, whether follicular fluid‐derived exosomes of POF patients have potential value in evaluating disease severity remains unknown. In the present study, we were surprised to find that nest granulosa cell‐specific knock‐in of miR‐3061^+/−^/AMH‐Cre^+/−^ in microRNA‐3061 transgenic mice resulted in the downregulated expression of the transcription factor PAX7 and Wnt/Ca^2+^ signalling pathway genes; this phenomenon was also manifested in the follicular fluid exosomes of POF patients. Our results preliminarily verified the experimental results in model animals, indicating that both PAX7 and the Wnt/Ca^2+^ signalling pathway were essential for the maintenance of normal physiological and biochemical functions of OGCs and that the mRNA expression levels of the aforementioned genes were significantly downregulated in the follicular fluid exosomes of patients during the pathogenesis of POF. The expression levels of Wnt5a, Fzd1, Camk2a and Pax7 genes in the PAX7 and Wnt/Ca^2+^ signalling pathways were also correlated with sex hormone concentrations in blood. We posit that our study will in the future provide a valuable reference for the accurate, noninvasive diagnosis of POF in women.

**FIGURE 8:**
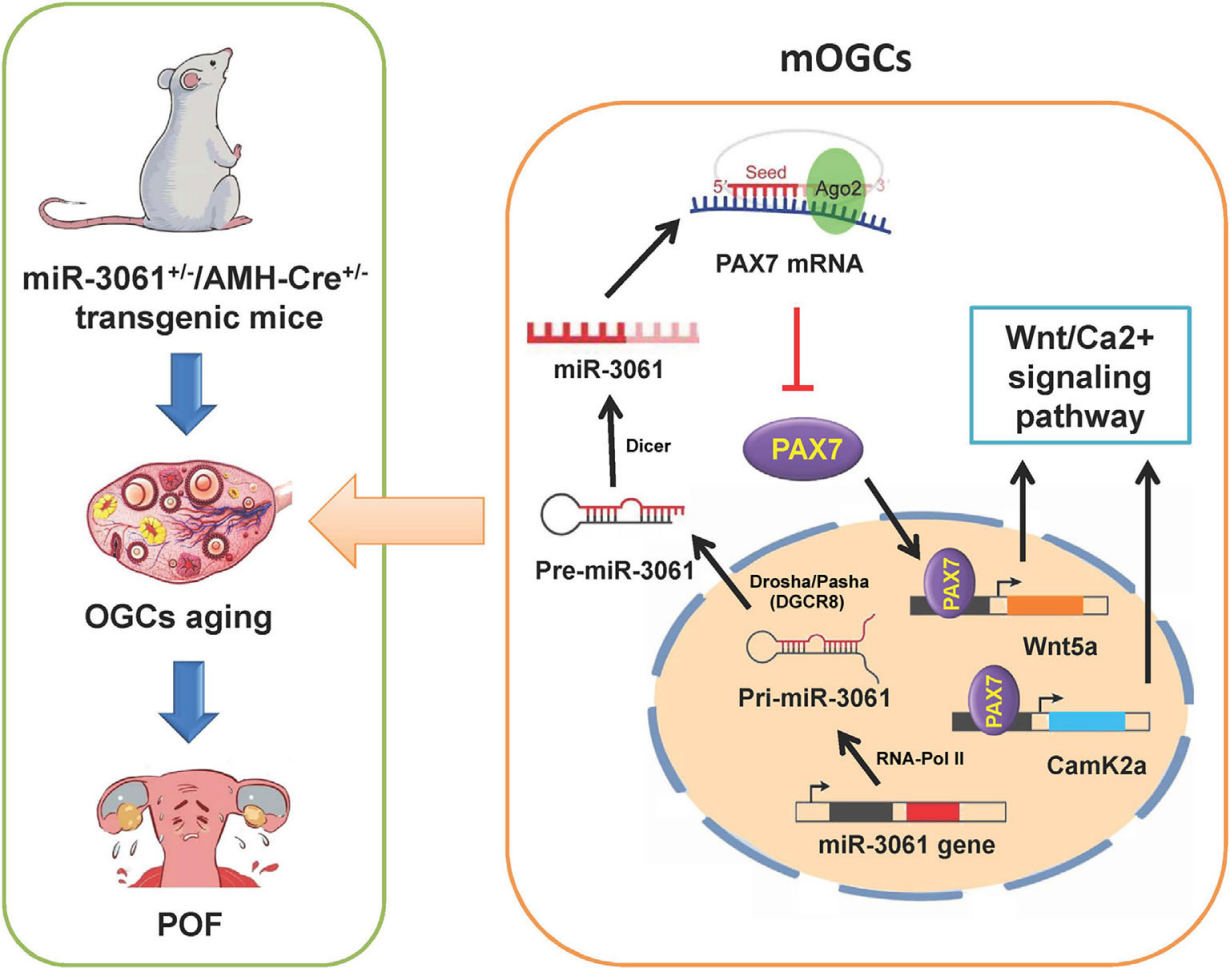
MicroRNA‐3061 downregulates the expression of PAX7/Wnt/Ca^2+^ signalling axis genes to induce premature ovarian failure in mice.

In conclusion, the present study confirmed that overexpression of microRNA‐3061 (miR‐3061–5p) negatively regulated the expression of transcription factor PAX7 and downregulated the transcriptional activity and expression levels of downstream Wnt/Ca^2+^ signalling pathway genes, inducing cell‐cycle arrest, retarding the proliferation rate of mOGCs, and thus ultimately inducing POF in mice (Figure 8).

## AUTHOR CONTRIBUTIONS

Te Liu, Yichao Wen, Zeyu Cui, Haiyang Chen and Jiajia Lin performed the majority of the experiments in the study. Jianghong Xu, Danping Chen, Ying Zhu, Zhihua Yu, Chunxia Wang and Bimeng Zhang contributed to the analysis of experimental data. Te Liu, Chunxia Wang and Bimeng Zhang contributed to the study design, manuscript writing and provided experimental funding support. All authors read and approved the final manuscript.

## FUNDING INFORMATION

This work was supported by grant from the National Natural Science Foundation of China (No. 82274569 and 81973899) and Clinical Research Special Fund of Shanghai Municipal Health Commission (202240223).

## CONFLICT OF INTEREST STATEMENT

The authors declare no conflicts of interest.

## Supporting information


**Supplementary Table S1.** Results of RNA‐seq.

## Data Availability

The data that support the findings of this study are available from the corresponding author upon reasonable request.
